# Indiana Association of Veterinary Graduates

**Published:** 1893-03

**Authors:** W. L. Williams, J. E. Cloud

**Affiliations:** President; Secretary


*Indiana Association of Veterinary Graduates.*—The annual meeting of the Indiana Veterinary Association was held at the New Clinton House, Kokomo, Ind. January 10th and 11th, 1893.

The meeting was convened at 1.30 P.M., the 10th, the president W. L. Williams in the chair. There were present, Drs. F. A. Balser, C. N. Bell, L. N. Roberts, M. J. Schaeffer, W. L. Williams, J. N. Honan, J. C. Rodger, A. J. Thompson, T. B. Pote, A. B. Carter, O. L. Boor, P. Justice, J. W. Watson, and C. M. Stull.

The minutes of the previous meeting were read and approved.

The Treasurer, Dr. Galbraith, being absent on account of sickness, presented his report through the Secretary, showing the association to be in improved financial condittion.

The following named veterinarians were proposed for membership: J. W. Reed, R. L. Chamberlain Fred. Braggington, E. T. L. York, J. D. Sturn, T. B. Pote,

A. B. Carter, O. L. Boor, P. Justice, J. W. Watson, C. M. Stull, S. Fitch and O. G. Whitestine. On motion of Dr. Roberts, the Secretary was instructed to cast the ballot of the association in favor of all the applicants and they were announced duly elected.

A communication was presented from Dr. E. F. Diggs, tendering his resignation as a member on account of engaging in the manufacture and sale of secret "specific" medicines, which upon motion of Dr. Roberts, was accepted and the Secretary instructed to notify Dr. Diggs accordingly.

On motion of Drs. Bell and Roberts, a committee of three, consisting of the President, Vice-President and Secretary, was created to revise the constition and by-laws, with power to print the same at the expense of the association and have it ready for presentation to the association at its next meeting.

Dr. Bell then presented his paper on "Amputation of the Penis," in which he detailed several cases of more than ordinary interest, occurring in his own practice. The paper was followed by an interesting discussion participated in by almost all members present, during the course of which many instructive cases of this operation were related and numerous useful suggestions brought out, chief of which were that direct amputation through urethra and corpus cavernosum was likely to be followed by urethral stricture, and that this was preventable by dissecting out the urethra, and allowing it to project beyond the corpus cavernosum, etc.

The association then adjourned to meet at the Elks Hall to which session the general public had been invited to be present, but owing to very inclement weather the audience proved small though appreciative. The Vice-President, Dr Bell presided, and the President delivered his annual address entitled "The Relation of the Veterinarian to the Public," in which the speaker showed that these relations had become so important that the veterinarian constituted an essential factor in the progress prosperity and health of the people. This was followed by a well prepared paper by Dr. A. G. Thompson entitled, ' Homeopathy in Relation to Veterinary Science," after which the meeting adjourned to the Clinton House and fully discussed it.

The president then called the attention of the association to the approaching International Veterinary Congress to be held at Chicago, September, 1893, and urged that the veterinarians of Indiana should fully awaken to their apportunities and responsibilities in this which will doubtless prove the most important veterinary meeting so far held in America.

We detailed the many courtesies extended to visiting veterinarians at Boston, last September and suggested that equally good entertainment should be provided at Chicago, for all attending veterinarians.

On motion of Dr. Thompson, the president appointed Drs. Boor, Stull and Balser a committee to confer, and act with like committees already appointed by the state associations of Illinois and Iowa for making arrangements and providing entertainment for the international meeting. The association then proceeded with the election of officers for the ensuing year with the following results: President, W. L. Williams, Lafayette; 1st, 2nd, and 3rd Vice-Presidents, Drs. C. F. Bell, C. M. Stull, and G W. Roberts, respectively; Secretary, J. E. Cloud, Richmond; Treasurer, F. A. Balser, Newcastle; Trustees, A. J. Thompson, T. B. Pote, O. L. Boor, M. J. Schaffer and J. W. Watson.

On motion of Dr. Bell it was decided to hold the next meeting of the association at Newcastle, in July.

The association then adjourned to meet at 8 A. M., the 11th, when through the courtesy of Dr. Bell the members visited the Kokomo Plate Glass Works. one of the largest concerns of the kind extant, where the members were shown hurriedly the various departments in active operation.

The members then proceeded to Dr. Bell's infirmary, where Dr. Balser demonstrated in a neat manner the method of castrating crytorchid horses, after which the meeting was called to order at the New Clinton House and an interesting paper presented by Dr. Honan, on "Inversion of the Uterus," which was followed by a spirited discussion engaged in generally by members present, the discussion turning largely upon the question of the necessity for retaining sutures truss or pessary after replacement, the general opinion seeming to be that they were at least useless in most cases especially if care be taken to straighten out the invaginations of corona and properly replace them and then withdraw the arm slowly and cautiously after the lapse of several minutes after replacement.

A paper by Dr. W. B. Wallace was then presented by Dr. Bell, entitled "Fracture of the Os Pedis," in which a rather remarkable ease was reported which according to the views of most members present was due to osted porosis.

On motion of Dr. Stull it was determined to secure necessary funds for assisting in entertainment of international veterinary congress by subscription.

Hearty good will prevailed throughout the meeting, the papers submitted were very good, the discussions were unusually spirited vnd instructive, the attendance larger than at any previous meeting and the accessions to membership also exceeded in number and value those of any prior meeting, so that on the whole those present felt greatly encouraged and considered this the best meeting in the history of the Indiana Veterinary Association. The meeting was then adjourned to convene in New Castle, early in July.

W. L. Williams, *President.* J. E. Cloud, *Secretary.*

